# Troubles des conduites alimentaires et tempérament cyclothymique: étude transversale à propos de 107 étudiants Tunisiens

**DOI:** 10.11604/pamj.2014.18.117.2936

**Published:** 2014-06-05

**Authors:** Masmoudi Jaweher, Trabelsi Sonda, Ouali Uta, Feki Inès, Sallemi Rim, Baati Imene, Jaoua Abdelaziz

**Affiliations:** 1 Service de Psychiatrie A, CHU Hédi Chaker, 3029 Sfax, Tunisie

**Keywords:** Trouble des conduites alimentaires, tempérament, étudiant, Eating disorder, temperament, student

## Abstract

**Introduction:**

Les objectifs de notre étude ont été d'estimer la prévalence des troubles des conduites alimentaires (TCA) chez les jeunes tunisiens et étudier la relation entre le tempérament cyclothymique et les TCA.

**Méthodes:**

Nous avons ainsi mené une étude transversale descriptive et analytique. Elle a concerné 107 étudiants de l'Institut de Presse et des Sciences de l'Information de la Manouba, Tunisie. Pour l’évaluation des TCA, nous avons procédé par la passation de l'auto questionnaire EAT 40, dans sa version validée en Tunisie. C'est l'outil le plus utilisé pour le dépistage des TCA dans le monde. Pour l’évaluation du tempérament cyclothymique, nous avons utilisé le TEMPS A dans sa version arabe validée. Une fiche épidémiologique associée a permis de recueillir quelques facteurs sociodémographiques et hygiéno-diététiques.

**Résultats:**

La prévalence des troubles de conduites alimentaires a été de 24,3%. Le pourcentage des étudiants ayant un score de tempérament cyclothymique ≥14 a été de 37,4%. Une association a été trouvée entre les troubles de conduites alimentaires et le tempérament affectif cyclothymique que ce soit selon l'approche dimensionnelle (p=0,005) ou selon celle catégorielle (p=0,046). Le tempérament cyclothymique multiplie par deux le risque de développer un TCA chez les étudiants de sexe féminin (p=0,04).

**Conclusion:**

Es TCA sont fréquents chez nos étudiants particulièrement de sexe féminin. De plus, la présence d'un tempérament cyclothymique associé permettrait de suspecter doublement une appartenance au spectre bipolaire et devrait conduire à une attention particulière de la part du clinicien pour définir au mieux les stratégies thérapeutiques.

## Introduction

Les troubles des conduites alimentaires (TCA) sont représentés par deux syndromes sévères, la boulimie et l'anorexie, entre lesquelles il existe un continuum clinique plus large constitué par des syndromes cliniques partiels et/ou des conduites de perte de poids connues sous le nom des troubles des conduites alimentaires non spécifiés (TCA NS). Ces troubles, longtemps méconnus, semblent devenir de plus en plus fréquents de part le monde [1–3]. De plus, ils évoluent dans la moitié des cas vers un syndrome complet [4–6].

Le souci de minceur insistant, envahissant, devient parfois obsédant surtout chez les adolescents et les jeunes adultes qui attribuent une grande importance à leur apparence. Ce qui pourrait conduire, chez les plus vulnérables, au développement des TCA [7, 8]. Cette vulnérabilité peut être de plusieurs ordres: psychologique, biologique, ou encore sociologique.

Le tempérament, base biologique de la personnalité, défini comme des dimensions relativement stables de l'organisme, signe les traits particuliers de réactions de chaque personne, tels que le niveau d’énergie et les caractéristiques du comportement. Le tempérament est considéré comme un facteur prédictif de survenue de nombreux troubles psychiatriques [9, 10]. Dans ce cadre, il peut correspondre à un facteur de vulnérabilité d'ordre biologique, contribuant à la survenue de TCA.

Le tempérament cyclothymique (TC) a été celui le plus associé aux TCA en littérature, qui demeure très réduite, concernant cet aspect [11–13]. Les objectifs de notre travail ont été d'estimer la prévalence des TCA NS, du TC chez les étudiants tunisiens et d’étudier la relation entre le tempérament cyclothymique et les TCA NS.

## Méthodes

Il s'agit d'une enquête transversale, descriptive et analytique, qui s'est déroulée à l'Institut de Presse et des Sciences de l'Information de Manouba(Tunisie). Elle a été réalisée le 6 et le 7 Décembre 2009 au cours des séances d'enseignement dirigé. Nous avons inclus dans ce travail au hasard 107 étudiants ayant eu des cours d'enseignement dirigé les après midi des 6 et du 7 décembre 2009. Ces étudiants ont été âgés de plus de 20 ans et ont donné leur consentement libre et éclairé. Nous avons exclu de ce travail les étudiants qui ont refusé de participer à l’étude et âgés de moins de 20 ans. La passation des fiches s'est faite pendant une heure d'enseignement, après l'obtention préalable de l'accord du directeur de l’établissement. Notre population a été d’âge moyen de 22,7 ans (ET=1,53). La majorité de notre échantillon (77,6%) a été de sexe féminin, soit un sex-ratio H/F= 0,29.

Il s'agit d'une enquête transversale, descriptive et analytique, qui s'est déroulée à l'Institut de Presse et des Sciences de l'Information de Manouba(Tunisie). Elle a été réalisée le 6 et le 7 Décembre 2009 au cours des séances d'enseignement dirigé. Nous avons inclus dans ce travail au hasard 107 étudiants ayant eu des cours d'enseignement dirigé les après midi des 6 et du 7 décembre 2009. Ces étudiants ont été âgés de plus de 20 ans et ont donné leur consentement libre et éclairé. Nous avons exclu de ce travail les étudiants qui ont refusé de participer à l’étude et âgés de moins de 20 ans. La passation des fiches s'est faite pendant une heure d'enseignement, après l'obtention préalable de l'accord du directeur de l’établissement. Notre population a été d’âge moyen de 22,7 ans (ET=1,53). La majorité de notre échantillon (77,6%) a été de sexe féminin, soit un sex-ratio H/F= 0,29.

### Instruments d’évaluation Eating Attitude Test dans sa version longue à 40 items (EAT40)

C'est un auto questionnaire développé par Garner et Garfinkel en 1979 [14]. Il est utilisé pour l’évaluation des attitudes alimentaires et le recueil des symptômes dans les populations ***non cliniques***. Il permet le ***dépistage*** des troubles des conduites alimentaires non spécifiés sans différenciation du type. Cette échelle a été validée en Tunisie en 2007. Le score seuil retenu a été de **30** [15].

### L’évaluation du tempérament affectif cyclothymique s'est faite grâce au TEMPS-A

(Temperament Evaluation of the Memphis, Pisa, Paris, and San Diego- Auto questionnaire): Nous avons utilisé les 21 items correspondant au tempérament cyclothymique dans sa version arabe telle que traduite et validée par Karam et al [16]. Un score seuil de 10 permet selon certains travaux de dépister le tempérament cyclothymique, alors qu'un score de 14 permet le diagnostic positif de ce tempérament [1]. Nous avons choisi un score de 14 pour définir les sous groupes: avec /sans tempérament cyclothymique.

### Etude statistique

Nous avons utilisé le logiciel SPSS dans sa 11ème version pour la saisie et l'analyse des données. Nous avons divisé la population en deux sous-groupes: *TCA*+ (groupe qui présente des troubles des conduites alimentaires) et *TCA*-(groupe qui ne présente pas des troubles des conduites alimentaires). Dans l’évaluation du lien entre les TCA et le TC, nous avons utilisé d'abord une approche dimensionnelle grâce à la comparaison des scores moyens du tempérament dans les deux sous-groupes (TCA+ versus TCA-). Ensuite nous avons complété cette évaluation par une approche catégorielle. Pour l’étude de la relation entre les variables catégorielles, le test de Khi 2 de Pearson et le test exact de Fisher ont été utilisés. Pour l’étude de la relation entre les variables catégorielles et les variables quantitatives, le test d'ANOVA a été utilisé. Le seuil de signification a été fixé à 5%.

## Résultats

### Prévalence des troubles de conduites alimentaires (TCA)

**Figure 1:**
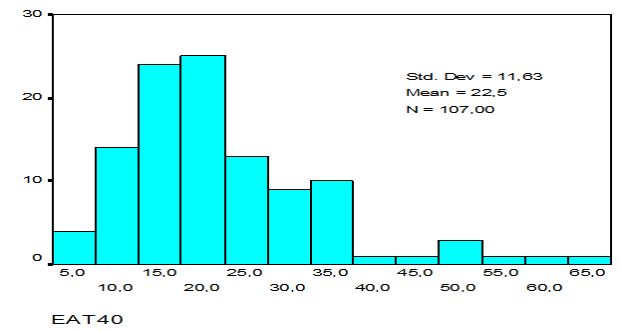
Distribution des scores des TCA

Le score des TCA à l’échelle EAT 40 a varié de 6 à 63. Le score moyen était de 22,47 (ET=11,6). Ces scores ont suivi une distribution Gaussienne (Figure 1). Vingt six étudiants ont eu un score supérieur à 30, soit une prévalence des troubles de conduites alimentaires de 24,3%. Ces troubles ont concerné significativement plus le groupe féminin que celui masculin (28,9% versus 9%; p=0,038; RR=1,3).

### Le tempérament cyclothymique Prévalence

**Figure 2:**
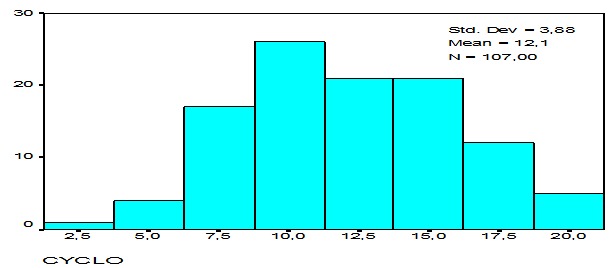
Distribution des scores du tempérament cyclothymique

Le score du tempérament cyclothymique a varié de 3 à 21. Le score moyen a été de 12,1 (ET= 3,8). La distribution de ces scores a suivi une courbe Gaussienne (Figure 2). Environ le tiers de la population (37,4%) ont eu un score ≥ 14.

### Relation entre le tempérament cyclothymique et les TCA

Selon l'approche dimensionnelle, le score moyen du tempérament cyclothymique a été significativement plus élevé chez le groupe présentant des TCA par rapport à celui sans TCA (13,88; ET=3,33 versus 11,46; ET=3,87: p=0,005). De même cette relation entre les TCA et le tempérament affectif cyclothymique a été confirmée selon l'approche catégorielle (p=0,046) (Tableau 1).

**Tableau 1:** Relation entre TCA et tempérament cyclothymique selon l'approche catégorielle

	TCA+	TCA-	p
TC=14	14	26	0,046
TC < 14	12	55	

### Relation entre le tempérament cyclothymique, les TCA, et les paramètres sociodémographiques

Seule une relation significative a été trouvée avec le sexe féminin pour les sujets à la fois cyclothymiques et ayant des TCA (p=0,04) (Tableau 2). Prenant en considération ce facteur, parmi les étudiantes non cyclothymiques 20,4% présentent des troubles de conduites alimentaires. Alors que, parmi les étudiantes cyclothymiques 41,17% ont eu des TCA; soit un risque multiplié par 2 environ.

**Tableau 2:** Relation entre le tempérament cyclothymique, les TCA et le sexe

		TC		p
		OUI	NON	
**Sexe masculin**	TCA+	0	2	0,39
	TCA -	6	16	
	**Total**	**6**	**18**	**24**
**Sexe féminin**	TCA+	14	10	0,04
	TCA -	20	39	
	**Total**	**34**	**49**	**83**

## Discussion

### Prévalence des troubles de conduites alimentaires

Dans notre étude, la prévalence des troubles de conduites alimentaires non spécifiés a été estimée à 24,3%. Les TCA non spécifiés, dépassant les limites des deux troubles les plus graves et les plus anciens, l'anorexie et la boulimie, sont souvent mesurés sélectivement par l'EAT 40 ou encore par l'EAT 26 [14]. La prévalence moyennant ces deux outils dans des populations d'adolescents non cliniques se situe entre 5,5 et 28, 4% de part le monde [15, 17]. Les études antérieurement réalisées dans notre pays et sur des populations adolescentes non cliniques, rapportent des chiffres qui varient entre 13% et 28% [15, 18]. Alors qu'elles varient entre 11,4% et 15,4% dans d'autres pays arabes [17, 19].

Ainsi, les prévalences des TCA NS trouvées dans la littérature tunisienne, tout comme celle arabe, se rapprochent de la littérature occidentale. Ces troubles réputés être différents en fonction des races, des ethnies et des cultures, en l'occurrence, plus fréquents en occident, tendraient actuellement à se répartir uniformément dans le monde entier [20–24]. De plus, et malgré les difficultés méthodologiques pour approcher la prévalence des TCA (nécessité de longues périodes pour mettre en évidence des changements modestes, l’évolution des techniques de mesure et des tenues de registre, des comportements des populations, etc.), la majorité des auteurs est d'accord sur une augmentation sensible de l'incidence actuelle des TCA chez les 15-24 ans [13, 25]. Cette augmentation de l'incidence et de la prévalence des TCA a été expliquée par le fait que ces troubles pourraient être considérés comme un nouveau mode préférentiel d'expression symptomatique des difficultés psychologiques à l'adolescence, rejoignant en cela d'autres troubles, notamment les conduites addictives (toxicomanie, alcoolisme). L'augmentation parallèle des TCA et des conduites addictives serait un argument supplémentaire en faveur de la pertinence du concept d'addiction dans les TCA quelle que soit l'approche épidémiologique, comportementale, biologique ou psychodynamique qui l'argumente [26, 27].

Des études transculturelles, mettant en évidence la montée d'incidence des TCA dans des pays en voie de développement où les préoccupations pour la minceur seraient moindres, ont fait pointer l'influence des médias à diffusion planétaire dans cette généralisation des troubles. Dans une perspective plus globale, ont été évoquées la mutation accélérée de l'identité et du rôle de la femme, ainsi que la quête de performance que la mondialisation provoquerait dans ces sociétés [28, 29]. Néanmoins, la question de savoir pourquoi seuls certains sujets seront sensibles à ces changements et seront amenés à développer ces troubles (vulnérabilité génétique, biologique, psychologique, sociologique,..) reste entière.

D'un autre angle, les TCA ont été pour longtemps considérés comme une pathologie essentiellement féminine [5, 6, 30]. Nos résultats vont également dans ce sens. Bailly explique cette prédominance féminine par le rôle fondamental des transformations pubertaires tant physiques que psychologiques, et le fait que la « problématique narcissique », centrale dans les TCA, se joue chez la femme au niveau de l'apparence, expliquant ainsi l'utilisation défensive du corps [6, 30]. Dans les études menées sur des populations non cliniques, les hommes ayant des TCA, présentent également plus de syndromes partiels que de syndromes complets [30].

### Tempérament cyclothymique: prévalence, lien avec les TCA et connexion bipolaire

Dans notre population, la prévalence du TC a été estimée à 37,4%. Ce chiffre est élevé par rapport à la littérature qui situe une prévalence des cyclothymiques entre 3 et 6%, dans des populations cliniques [16, 31, 32]. Cette prévalence élevée peut être expliquée par le fait que notre population a été majoritairement de sexe féminin. En effet, certaines études rapportent une prédominance féminine de la cyclothymie [33]. De plus, Certains auteurs rapportent une fréquence relativement élevée du tempérament cyclothymique dans la population générale, donc non clinique, mais très peu diagnostiquée spontanément par le clinicien [16, 31]. Notre échantillon est fait de jeunes étudiants non cliniques.

De plus, nous avons trouvé une association significative entre le tempérament cyclothymique et les TCA NS (p=0,048) rejoignant ainsi les données de la littérature [11, 34, 35]. Dans notre étude, une association significative a été trouvée entre le sexe féminin, les TCA NS et le tempérament cyclothymique. En effet, d'une part, le sexe féminin multiplie le risque d'avoir un TCA NS par 1,3. D'autre part, la présence de tempérament cyclothymique en plus du sexe féminin fait augmenter le risque à 2. Il est connu que les TCA, ainsi que la cyclothymie, sont plus fréquents chez le sexe féminin [30, 32]. La présence d'un tempérament cyclothymique chez une femme paraît être potentialisateur des TCA, comme soulignent Hantouche et Signoretta [11, 34].

Au cours de ces dernières années, quelques auteurs se sont intéressés à l'association TCA et tempérament cyclothymique évalué par l’échelle TEMPS: Signoretta et al. (2005) ont étudié le tempérament affectif (TEMPS version Italienne) et les difficultés du comportement émotionnel (Emotional and Behavioral Checklist in Infancy, Childhood and Adolescence) dans un échantillon non clinique de 1010 jeunes étudiants (518 garçons et 492 filles). Ils ont montré qu'une disposition cyclothymique a été plus fréquemment associée à l'anxiété, aux troubles de sommeil, à la sensibilité à la séparation et aux troubles alimentaires pour le sexe féminin, et à des comportements antisociaux et agressifs pour le sexe masculin [12]. Ainsi, nos résultats ont été corroborés, spécifiquement pour le sexe féminin; Ramacciotti et al. (2004) ont étudié les tempéraments affectifs dans un échantillon de patients ayant des TCA selon le SCID, en le comparant à une population normale. L'instrument de l’étude a été le TEMPS-I. Le groupe de patients ayant une anorexie mentale n'a pas été corrélé à un tempérament affectif donné. En revanche, 24% du groupe des boulimiques ont eu un tempérament affectif dominant. La corrélation la plus significative a concerné le tempérament cyclothymique [34]; Une autre étude récente, réalisée par Amann et al. (2009), a essayé d’évaluer les profils tempéramentaux chez 213 patients souffrant d'obésité morbide. Le groupe des obèses a montré beaucoup plus de co-morbidité dépressive, et de tempérament anormal, comparativement au groupe témoin. Les tempéraments cyclothymique, irritable et anxieux ont été hautement corrélés à l'obésité [11].

### Connexion avec la bipolarité

D'un autre angle, les notions de la cyclothymie et du tempérament cyclothymique, même si voisines dans leur expression clinique, sont différentes au niveau nosographique. La cyclothymie, faisant partie des troubles bipolaires dans le sens d'un trouble (DSM IV) exige une altération du fonctionnement socio-familial ou professionnel, une souffrance du patient et de sa famille. Alors qu'une cyclothymie dans le sens tempéramental concerne plutôt la base biologique de la personnalité [36]. Akiskal et Mallaya considèrent les tempéraments comme des manifestations atténuées du trouble bipolaire lui-même [37–40]. Plus récemment, les *« traits » cyclothymiques ont été conçus comme liant un continuum polygénique entre le tempérament « excessif » et le « désordre » bipolaire* [37, 41].

D'un point de vue plus large, et pour certains auteurs, les traits cyclothymiques et les TCA semblent appartenir au spectre bipolaire. En effet, certaines études récentes consacrées aux addictions comportementales montrent des similitudes avec les phénomènes obsessionnels compulsifs (contrainte compulsive) et bipolaires (colère impulsive et prise de risque). Parmi ces addictions, les TCA sont sans doute les plus connues. Ces études suggèrent leur appartenance au spectre bipolaire [31, 32].

Perugi G et al., ont tenté de redéfinir le spectre de la bipolarité. Ils y incluent une disposition cyclothymique - anxieuse sensible, une réactivité de l'humeur et une sensibilité interpersonnelle ainsi qu'un défaut de contrôle des impulsions et les troubles de conduites alimentaires [7, 8]. L'association de ces deux conditions cliniques pourrait être en faveur de leur appartenance au spectre bipolaire.

## Conclusion

Nos résultats corroborent la tendance mondiale à une répartition de plus en plus homogène des TCA, et surtout des TCA NS. Ces troubles ont été associés au tempérament cyclothymique, notamment pour le sexe féminin. Plusieurs auteurs stipulent que les traits cyclothymiques paraissent lier un continuum polygénique entre le tempérament excessif et le désordre bipolaire et que la présence d'un tempérament cyclothymique est un marqueur robuste de bipolarité. D'autres auteurs tendent à inclure, de même, les troubles de conduites alimentaires dans le spectre bipolaire. Le fait que ces deux conditions soient liées pourrait constituer une sorte de validation externe d'appartenance de ces deux entités au même spectre, bipolaire en l'occurrence. Ce qui pourrait inciter le clinicien à dépister ce tempérament chez les sujets avec un TCA NS. Cette hypothèse demande à être confirmée par des études prospectives de longue durée, et avec la tenue de registre de données. En fait, les implications thérapeutiques sont importantes notamment concernant l'utilisation des thymorégulateurs et leur place dans les futures stratégies thérapeutiques.

## References

[1] Mechri A, Kerkeni N, Touati I (2011). Association between cyclothymic temperament and clinical predictors of bipolarity in recurrent depressive patients. Journal of affective disorders..

[2] Mintz LB, Mso'Halloran (2000). The Eating Attitude Test: validation with DSMIV eating disorder criteria. J Personal Assess.

[3] Paxton S, Diggens J (1997). Avoïdance coping, binge eating, and depression: An examination of the escape theory of eating. Int J Eat Disord..

[4] Bunnel DW, Shenker IR, Nussboum MP (1995). Subclinical versus formal eating disorder: differentiating psychological features. Int J Eant Disord..

[5] Herzog DB, Hokins JD (1993). Afollow up study of 33 sub diagnostic eating disorder women. Int J Eat Disord..

[6] Kerremans A, Claes L, Bijttebier P (2010). Disordered eating in adolescent males and females: Associations with temperament, emotional and behavioral problems and perceived self-competence. Personality and Individual Differences..

[7] Perugi G, Passino MC, Akiskal KK, Kaprinis S, Akiskal HS (2006). Bulimia nervosa in atypical depression: the mediating role of cyclothymic temperament. J Affect Disord..

[8] Perugi G, Akiskal HS (2002). The soft bipolar spectrum redefined: focus on the cyclothymic, anxious-sensitive, impulse-dyscontrol, and binge-eating connection in bipolar II and related conditions. Psychiatr Clin North Am..

[9] Akiskal HS (1988). Personality as mediating variable in the pathogenesis of mood disorders: implication for theory research and prevention.

[10] Strelau J (1983). Temperament, Personnality and Arousal.

[11] Amann B, Mergl R, Torrent C (2009). Abnormal temperament in patients with morbid obesity seeking surgical treatment. J Affect Disord..

[12] Signoretta S, Maremmani I, Liguori A (2005). Affective temperament traits measured by TEMPS-I and emotional-behavioral problems in clinically-well children, adolescents, and young adults. J Affect Disord..

[13] Van Hoeken D, Seidell J, Hoek HW, Treasure J, Schmidt U, Van Furth E (2003). Epidemiology. Handbook of eating disorders.

[14] Guelfi JD (1997). L’évaluation clinique standardisée en psychiatrie.

[15] Bouhlel S (2003). Troubles des conduites alimentaires, dépression et alexithymie chez une population d’étudiants en médecine: Prévalence et corrélation.

[16] Karam E, Mneimneh Z, Salamoun M, Akiskal K, Akiskal S (2005). Psychometric properties of the Lebanese-Arabic TEMPS-A: A national epidemiologic study. J Aff Disor.

[17] Nasser M (1994). Screening for abnormal eating attitudes in a population of Egyptian secondary school girls. Soc Psychiatry Epidemiol..

[18] Masmoudi J, Trabelsi S, Elleuch E, Jaoua A (2008). Les troubles des conduites alimentaires pendant et en dehors du ramadan. Journal de thérapie comportementale et cognitive..

[19] Al-Subaie A (2000). Some correlates of dieting behaviour in Saudi schoolgirls. Int J Eat Disord..

[20] Casper RC, Offer D (1990). Weight and dieting concerns in adolescents; fashion or symptom?. Pediatrics..

[21] Kreipe R, Dukarm C (1999). Eating disorders in adolescent and older children. Am Acad Pediatrics..

[22] le Grange D, Telch CF, Tibbs J (1998). Eating attitudes and behaviours in 1435 South African Caucasian and non-Caucasian college students. Am J Psychiatry..

[23] Lippincott JA, Hwang HS (1999). On cultural similarities in attitudes toward eating of women students in Pennsylvania and south Korea. Psychol Rep..

[24] Lunner K, Werthem EH, Thompson JK (2000). A cross-cultural examination of weight- related teasing, body image, and eating disturbance in Swedish and Australian samples. Int J Eat Disord.

[25] Lucas AR, Crowson CS, O'Fallon WM, Melton L (1999). The ups and downs of anorexia nervosa. Int J Eat Disord..

[26] Corcos M, Nezelof S, Bizouard P, Girardon N, Jeammet PH (2000). Pertinence du concept d'addiction dans les TCA. Ann Med Int Addiction.

[27] Davis C, Claridges G (1998). The eating disorders as addiction: a psychological perspective. Add Behav..

[28] Léonard T, Foulon C, Guelfi JD (2005). Troubles du comportement alimentaire chez l'adulte.

[29] Nasser M, Katzman M, Treasure J, Schmidt U, Van Furth E (2003). Sociocultural theories of eating disorders: an evolution in thought. Handbook of eating disorders.

[30] Olivardia R, Pope H, Mangrweth B (1995). Eating Disorders in college Men. Am J Psychiatry..

[31] Hantouche E (2006). “Troubles Bipolaires, Obsessions et Compulsions”.

[32] Hantouche E, Regis Balin (2008). La Cyclothymie pour le Meilleur et pour le Pire.

[33] Akiskal HS (1995). Le spectre bipolaire: acquisitions et perspectives cliniques. L'Encéphale..

[34] Ramacciotti CE, Paoli RA, Ciapparelli A, Marcacci G, Placidi GE, Dell'Osso L, Garfinkel PE (2004). Affective temperament in the eating disorders. Eat Weight Disord..

[35] Perugi G, Akiskal HS (2002). The soft bipolar spectrum redefined: focus on the cyclothymic, anxious-sensitive, impulse-dyscontrol, and binge-eating connection in bipolar II and related conditions. Psychiatr Clin North Am..

[36] Hantouche EG, Akiskal HS (2002). Les tempéraments affectifs.

[37] Akiskal HS, Bourgeois M (2000). Re-evaluating the prevalence of and diagnostic composition within thebroad clinical spectrum of bipolar disorders. J Affect Disord..

[38] Akiskal HS, Mallya G (1987). Criteria for the soft bipolar spectrum: treatment implication. Psychopharmacologybulletin..

[39] Hantouche EG, Akiskal HS (1997). Outils d’évaluation des tempéraments affectifs. Encephale..

[40] Van Den Bulke D, Henry C (2005). Intérêt de l'analyse du tempérament chez les sujets bipolaires. L'encéphale..

[41] Hantouche EG, Akiskal HS (2004). Connaître le spectre bipolaire dans sa globalité. Ann Med Psy..

